# An Unlikely Case of Benztropine Misuse in an Elderly Schizophrenic

**DOI:** 10.7759/cureus.13434

**Published:** 2021-02-18

**Authors:** Michael Esang, Ulziibat S Person, Odeyuwa O Izekor, Thien Kim Le, Donya Ahmadian

**Affiliations:** 1 Psychiatry, Clarion Psychiatric Center, Clarion, USA; 2 Psychiatry and Behavioral Sciences, Nassau University Medical Center, East Meadow, USA; 3 Psychiatry and Behavioral Sciences, American University of the Caribbean School of Medicine, Cupecoy, SXM

**Keywords:** benztropine, haloperidol, schizophrenia, anticholinergic misuse, geriatric, dopamine, muscarinic, gastrointestinal, urologic, complications

## Abstract

Although data on the prevalence of anticholinergic misuse is scarce, it has been reported among psychiatric patients. Anticholinergic drugs can act as potent indirect dopamine agonists in the limbic system, a mechanism that has been hypothesized to explain their misuse potential among patients. In psychiatric practice settings, the use of typical antipsychotics in conjunction with anticholinergics is common, with the latter mainly used to manage extrapyramidal side effects of the former. Haloperidol is a first-generation (typical) antipsychotic with weak anticholinergic properties that may sometimes be potentiated when it is used in combination with other anticholinergic medications. This combination can induce significant gastrointestinal hypomotility, constipation, and rarely even paralytic ileus. We present the case of a 67-year-old African American male with a history of schizophrenia, benign prostatic hyperplasia, and essential hypertension, who abruptly started misusing benztropine, without any prior history of a substance use disorder. This case highlights the importance of obtaining a detailed history when previously stable psychiatric patients develop acute physical symptoms. It also illustrates the importance of care coordination among care providers and the central role of the psychiatrist in the care of patients with medical comorbidities.

## Introduction

In psychiatry, anticholinergics have been used to manage extrapyramidal symptoms arising from the strong D2 receptor antagonism caused by antipsychotic medications. The extrapyramidal symptoms are believed to be due to dopamine antagonism in the nigrostriatal pathway of the brain. This pathway connects the substantia nigra pars compacta in the midbrain with the dorsal striatum in the forebrain. Anticholinergic medications help alleviate the neurologic effects of blocking D2 receptor activity in this pathway. These agents, however, can cause a variety of distressing peripheral and central adverse effects such as dry mouth, urinary disturbances, constipation, cognitive impairment, and in severe cases, delirium. The prevalence of anticholinergic misuse has been reported as 34% and occurs most commonly in psychiatric patients [1,2]. Benztropine, a medication often prescribed to treat Parkinson’s disease, is also utilized for the management of the extrapyramidal side effects of antipsychotic medications [3]. It has both anticholinergic and antihistaminic properties. It also binds the dopamine transporter (DAT) and inhibits the reuptake of dopamine (DA), resulting in increased dopaminergic neurotransmission in the mesolimbic (nucleus accumbens) and mesocortical systems. This is the critical element in mediating the reinforcing and psychostimulant effects of benztropine misuse [4]. Furthermore, benztropine has been found to improve some of the social and affective dysfunction seen in schizophrenia, perhaps providing additional incentives for its misuse [4]. Overuse of this medication subsequently increases the likelihood of anticholinergic side effects, which include constipation, urinary retention (M3 receptor blockade), decreased appetite, dry mouth, and dry skin, as well as drowsiness, confusion, delirium, and hallucinations [5]. Each anticholinergic medication that a patient takes may increase the risk of cognitive impairment by 46% over six years [6]. This review aims to shed light on the possibility of benztropine misuse, even in a stable elderly schizophrenic with no significant prior history of problems related to substance use. The authors highlight benztropine’s pharmacological and clinical profile and the most prominent drawbacks from its misuse, especially in the geriatric patient population.

## Case presentation

The patient was a 67-year-old African American male who had a psychiatric history of schizophrenia as well as a history of benign prostatic hyperplasia and essential hypertension. He had been receiving care for over 10 years at our ambulatory psychiatric center while on a monthly intramuscular injection of 50 mg of haloperidol decanoate. He had a past history of muscle stiffness from the haloperidol injection and this extrapyramidal symptom had been controlled with oral benztropine at 0.5 mg two times a day on an as-needed basis. During the course of his treatment, he developed hematochezia along with lower abdominal pain, constipation, and difficulty with urination, which was initially attributed to his enlarged prostate. However, a careful history by his psychiatrist subsequently revealed that he had been using Benztropine more than he was being prescribed. He admitted to taking more than double his prescribed dose per day as well as increasing doses over a period of months, as he had also been receiving this prescription from his gastroenterologist (up to a total of 5-8 mg per day in divided doses). He did not give any clear reason for taking higher doses of the benztropine and only stated that he was just taking his "side effect medication" as needed. The psychiatrist contacted his outpatient gastroenterologist and urologist, coordinating his care with these specialists. Extensive psychoeducation, over a period of multiple clinic visits, was provided to him focusing on his increased susceptibility to the anticholinergic effects of benztropine. It was explained that his advanced age and history of benign prostatic hyperplasia placed him at an elevated risk of anticholinergic complications. The benztropine dose was reduced and shorter duration prescriptions were provided to allow for increased monitoring and he began to use the medication as prescribed. His gastroenterologist and urologist had both ruled out a malignant neoplasm as the etiology of his presentation. These symptoms gradually resolved and he continued to receive psychiatric care regularly at our clinic without any further incidents.

## Discussion

Anticholinergic drugs bind to muscarinic receptors and block acetylcholine neurotransmission, which is involved in many major body functions including central nervous system (CNS) functions such as attention, learning, and memory mechanisms. Cholinergic transmission in the peripheral nervous system (PNS) is also involved in the basal functioning of humans such as urination, intestinal transit, or heart rhythm regulation [7].

When considering this patient, it should be noted that constipation is a minor side effect of haloperidol therapy because of its weak anticholinergic effect. In combination with anticholinergics such as benztropine, however, it can induce significant gastrointestinal hypomotility, constipation, and rarely, paralytic ileus [8]. In elderly patients with chronic medical comorbidities, iatrogenic anticholinergic effects can be overlooked as the potential culprit of these symptoms. Among the currently available antiparkinsonian anticholinergics, benztropine has the highest affinity for muscarinic acetylcholine receptors (mAChRs) [9]. Anticholinergic drugs can also act as potent indirect dopamine agonists in the limbic system [4]. Through the blockade of the muscarinic receptors, anticholinergic drugs inhibit dopamine reuptake and storage, accounting for the euphoric and hallucinogenic effects sometimes encountered with their use [10]. Although anticholinergics can exacerbate psychosis, they can also modestly improve negative symptoms of schizophrenia [9]. In accordance with these findings, patients with schizophrenia often report an activating effect at higher doses of anticholinergics, which sometimes can result in misuse [9]. This is an important clinical point and warrants further investigation, considering the limitations of existing therapies in combating the negative symptoms of schizophrenia spectrum disorders.

We reviewed relevant literature in order to explore clinical trends in the use of anticholinergic medications in treatment settings, and Table 1 summarizes our findings. Although our review was not systematic and was of limited scope, the cognitive burden of chronic anticholinergic use was immediately apparent and deserves to be mentioned.

**Table 1:** Summary of literature review findings on risks associated with anticholinergic use.

Sources	Summary of findings	Limitations
Ogino et al. (2014) [9]	Patients with schizophrenia often report an activating effect of higher doses of anticholinergics, which sometimes results in anticholinergic misuse.	This is the first study in its field to examine the effects of anticholinergic drug use with long‐acting injectable antipsychotics on cognitive function and safety in patients with schizophrenia.
Naja and Halaby (2017) [10]	Shifting, when possible, to second-generation antipsychotics could contribute to fewer extrapyramidal side effects, thereby limiting the need for anticholinergic drugs.	The majority of the data was related to trihexyphenidyl abuse although benztropine was noted as a drug that has significant abuse potential.
Lopez et al. (2019) [7]	Given the potential risk of irreversible cognitive effects in prolonged treatments, the efficacy and risks of anticholinergic drugs should be re-evaluated with longer treatment periods.	Drug scales used in this study did not account for drug interactions and comorbidities. The anticholinergic load was also underestimated. Furthermore, the results are not translatable worldwide as the scales had drugs that are not approved or commercialized in specific countries.
Ang et al. (2017) [11]	There was an inverse relationship between cumulative anticholinergic activity and cognition; those with higher medication anticholinergic burden had a deficit in their performance on cognitive tasks.	Medication adherence was not assessed in this study. Medication dose and frequency were un-adjusted for.
O’Reily et al. (2016) [12]	Anticholinergic burden significantly impacted reasoning and perception, which further weakened the ability of patients suffering from schizophrenia and schizoaffective disorder to benefit from psychosocial treatment programs.	Cross-sectional study, a limited sample size, and a limited duration in which patients were followed and assessed (three years).
Eum et al. (2017) [13]	Anticholinergic burden was inversely related to cognitive performance. This study explored the impact of cognitive impairment on a patient's ability to live and operate independently, their potential for occupational success, and other psychosocial factors.	As a cross-sectional study, the ability to establish causal relationships was therefore limited. The severity of disease/medication dose and duration of exposure were unaccounted for.

It is well established that chronic use of anticholinergic medications can increase the risk of cognitive impairment, particularly in geriatric patients. Ang et al. investigated the impact of anticholinergic use among individuals with schizophrenia [11]. The results of this study suggested an inverse relationship between cumulative anticholinergic activity and cognition, indicating that those with a higher medication anticholinergic burden had cognitive deficits [11]. In another study, the anticholinergic burden significantly impacted reasoning and perception in patients with schizophrenia, schizoaffective disorder, and psychotic bipolar illness [12,13]. Investigators were able to demonstrate the relationship between anticholinergic burden and negative outcomes of psychosocial treatment programs for patients with schizophrenia and schizoaffective disorder [12].

In spite of these drawbacks, geriatric prescriptions of anticholinergic drugs cannot always be avoided in clinical practice. For instance, a patient may be quite reluctant to switch to a different antipsychotic medication after multiple treatment failures with dire consequences. Where these medications remain crucial to keeping patients in remission, priority should be given to those with a mild anticholinergic load and those with more selectivity for receptors at their site of action. In addition, possible medication management problems should be addressed in view of the increased anticholinergic load associated with multiple anticholinergic agents [11]. Given the potential risk of irreversible cognitive effects in prolonged treatments, the efficacy and risks of anticholinergic drugs should be re-evaluated when treatment periods longer than three months are considered or anticipated [14]. When possible, switching to a second-generation antipsychotic could contribute to less extrapyramidal side effects, hence potentially obviating the need for anticholinergics. Although they may be safe to use over an extended period, anticholinergic effectiveness may diminish over time, and side effects such as sedation and cognitive impairment may worsen. Periodic trials of discontinuation could therefore be useful in justifying the need for continued use, especially in institutional settings where they are prescribed as adjuncts to antipsychotics [10].

## Conclusions

This case highlights the importance of obtaining a detailed history when previously stable psychiatric patients develop acute physical complaints. It also illustrates the importance of coordination among different care providers and specialists involved in the care of a patient. The central role of the psychiatrist in this regard cannot be overemphasized, especially in the management of patients with chronic and severe psychiatric illnesses. Even in elderly patients with no apparent prior history of a substance use disorder, anticholinergic medications can be misused leading to adverse health outcomes. Our review is limited in scope as we have reported only on a single individual. We, however, believe that the complex interrelationship among extra-pyramidal side effects of antipsychotics, anticholinergic use and their abuse potential, negative symptoms of schizophrenia spectrum disorders, and the negative effects of anticholinergics, deserves further investigation.
